# Compliant Intramedullary Stems for Joint Reconstruction

**DOI:** 10.1109/JTEHM.2024.3365305

**Published:** 2024-02-12

**Authors:** John A. Mccullough, Brandon T. Peterson, Alexander M. Upfill-Brown, Thomas J. Hardin, Jonathan B. Hopkins, Nelson F. Soohoo, Tyler R. Clites

**Affiliations:** Department of Mechanical and Aerospace EngineeringUniversity of California Los Angeles 8783 Los Angeles CA 90095 USA; David Geffen School of MedicineUniversity of California Los Angeles 8783 Los Angeles CA 90095 USA; Material, Physical, and Chemical Sciences CenterSandia National Laboratories 1105 Albuquerque NM 87123 USA; Department of Orthopaedic SurgeryUniversity of California Los Angeles 8783 Los Angeles CA 90095 USA

**Keywords:** Orthopaedics, aseptic loosening, compliant mechanisms, flexure, intramedullary stems, joint replacement

## Abstract

The longevity of current joint replacements is limited by aseptic loosening, which is the primary cause of non-infectious failure for hip, knee, and ankle arthroplasty. Aseptic loosening is typically caused either by osteolysis from particulate wear, or by high shear stresses at the bone-implant interface from over-constraint. Our objective was to demonstrate feasibility of a compliant intramedullary stem that eliminates over-constraint without generating particulate wear. The compliant stem is built around a compliant mechanism that permits rotation about a single axis. We first established several models to understand the relationship between mechanism geometry and implant performance under a given angular displacement and compressive load. We then used a neural network to identify a design space of geometries that would support an expected 100-year fatigue life inside the body. We additively manufactured one representative mechanism for each of three anatomic locations, and evaluated these prototypes on a KR-210 robot. The neural network predicts maximum stress and torsional stiffness with 2.69% and 4.08% error respectively, relative to finite element analysis data. We identified feasible design spaces for all three of the anatomic locations. Simulated peak stresses for the three stem prototypes were below the fatigue limit. Benchtop performance of all three prototypes was within design specifications. Our results demonstrate the feasibility of designing patient- and joint-specific compliant stems that address the root causes of aseptic loosening. Guided by these results, we expect the use of compliant intramedullary stems in joint reconstruction technology to increase implant lifetime.

## I. Introduction

OVER 2.5 million total joint arthroplasties (“replacements”) were performed in the United States in 2021 [1]. With an increasingly aging population, there is a growing demand for longer-lasting implants that improve mobility and reduce pain [2]. In a total joint arthroplasty, a damaged joint, such as a knee or hip, is replaced with synthetic parts that emulate the function of the healthy joint [3]. Unfortunately, joint replacements have a limited lifespan, with 15-year failure rates between 4 and 34 percent [4], [5], [6], [7]. The dominant non-infectious cause of failure of these implants is aseptic loosening, which describes loosening of the implant relative to the surrounding bones, without presence of infection or periprosthetic fracture. Aseptic loosening is responsible for between 23 and 64 percent of orthopaedic implant failures in hip, knee, and ankle replacements [8], [9], [10], [11], [12].

Intramedullary stems are a crucial component of joint arthroplasty, responsible for connecting the central joint-replacing structure to the surrounding bone. Conventional stem designs are composed of a rigid shaft that is bonded to the bone canal via cement, press-fit, or osseointegration [13], [14]. During gait, the leg sees complex 6-degree-of-freedom loads that include a non-trivial moment about the stem’s long axis [15]. If the reconstructed joint is constrained in any way that resists this axial rotation, the bulk of that load transfers to the stem, resulting in shear stress at the bone-implant interface [16]. To alleviate over-constraint, some joint replacement implants use polyethylene bearings that allow the joint to freely rotate about the stem’s axis [16], [17]. However, with repetitive loading over the lifetime of the implant, friction at the bearing surface releases polyethylene particles into the surrounding bone and tissue [16], [17], [18]. The presence of these foreign particulates in the bone triggers an inflammatory response that leads to bone resorption, called “osteolysis”. This weakens the bone and compromises mechanical fixation, eventually resulting in loosening of the implant [16], [19]. With a rigid stem, it is not possible to address over-constraint without inducing particulate wear, creating a tug-of-war between these two dominant failure modes.

**FIGURE 1.**
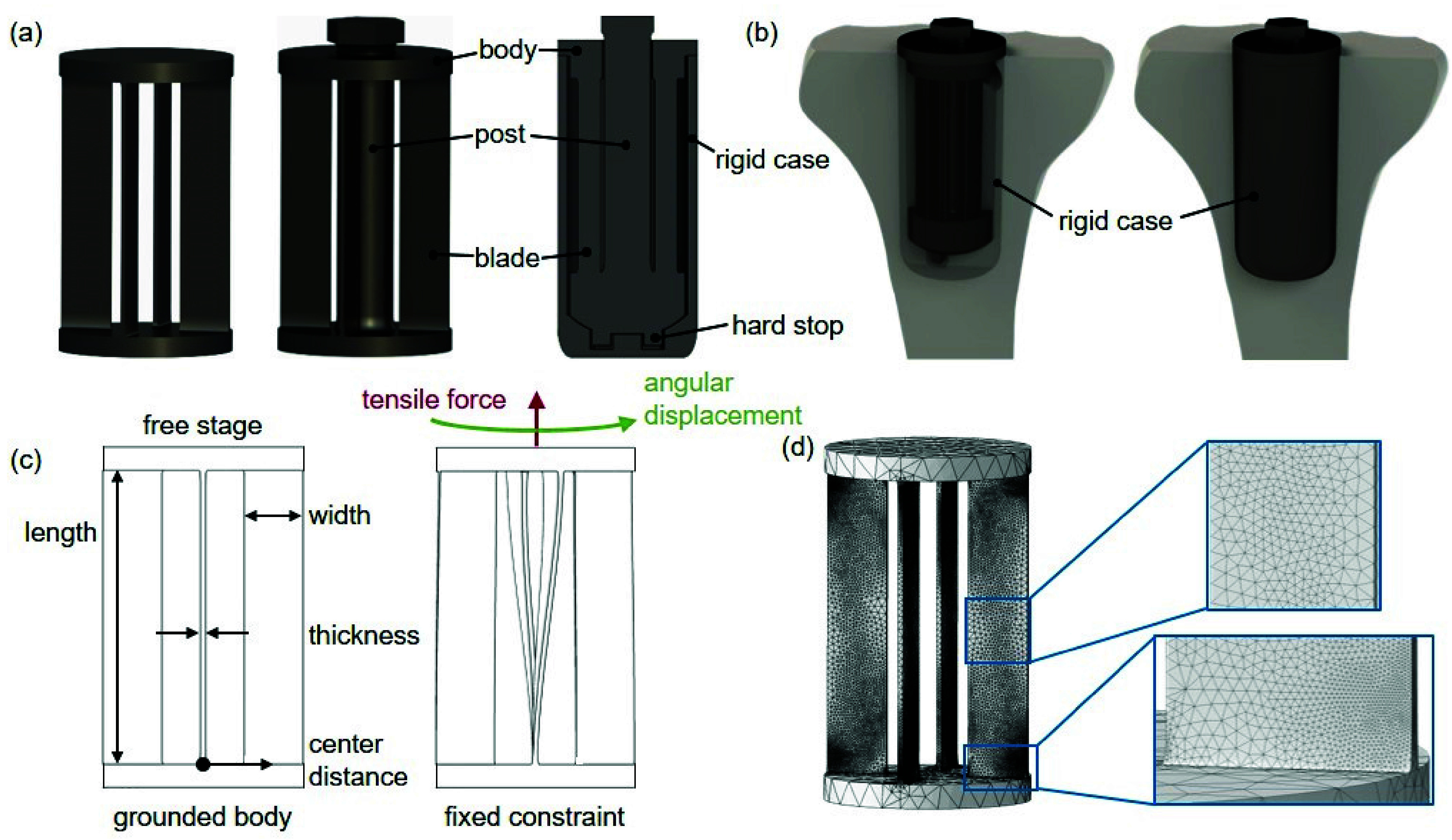
Compliant intramedullary stem design and finite element analysis (FEA). (a) Four-blade caged hinge with a central post to convert compression into tension, and a cross-sectional view of a caged hinge within a rigid case. (b) Compliant intramedullary stem with case (shown transparent) within the proximal tibia. (c) Schematic of the four-blade caged hinge used for FEA sweep, showing principal geometric parameters and FEA setup. (d) Mesh after three iterations of adaptive meshing.

To address both over-constraint and particulate wear with a single design, we have reimagined intramedullary stems to introduce compliance in rotation about their long axis. Our novel stem architecture consists of a compliant mechanism suspended within a rigid case (Fig 1). The compliant mechanism guides motion of one end of the stem (ideally where the stem connects to the artificial joint) with respect to the rigid case, about the stem’s long axis. The rigid case, which makes up the exterior of the stem, is designed to be anchored to the bone via standard bone-implant interfacing techniques such as cement or osseointegration; in this way, the stem has the potential to serve as a one-to-one replacement for conventional stems.

Compliant mechanisms are parts or systems of parts that achieve motion via deformation, rather than rubbing, rolling, or sliding. Mechanisms that guide motion in this way hold immense promise for orthopaedic implants because they can be designed to be inherently stable, can store and return energy, and are frictionless [20], [21], [22], [23], [24], [25], [26]. Especially in high-load environments, compliant mechanisms typically have longer lifespans than dynamically-equivalent traditional bearings, because they experience minimal wear. As such, compliant intramedullary stems have the potential to accommodate axial rotation without generating particulates, thereby mitigating osteolysis. Moreover, by nature of their innate compliance, we expect these stems to better accommodate joint motion, leading to lower stress both at the bone-implant interface and at the point of contact between polyethylene bearing components within the reconstructed joint.

The design space for compliant mechanisms is vast, and complex geometries are often required to achieve desired motions. The Freedom and Constraint Topologies (FACT) framework allows for rapid formulation of compliant mechanisms with desired mechanical properties [20], [21], [22], [26]. From the FACT-generated pool of potential mechanisms that provide one rotational degree of freedom, the caged hinge is unique in its ability to withstand high axial loads [27], [28]. A caged hinge is a mechanism with two parallel rigid bodies, a ground and a stage, connected by thin blades that extend radially from a single axis at their center. As the stage rotates relative to the ground, the thin blade members deform in torsion, creating a tunable compliance in rotation about the long axis of the mechanism. The caged hinge mechanism is well-suited to the needs of intramedullary stems, which require angular deformation about a single axis, under large compressive loads.

Reduction of the general caged-hinged geometry to a joint- and pathology-specific stem requires a design framework that predicts and prioritizes device mechanics under expected joint loads. In short, the ideal stem design will typically be the smallest mechanism that i) can withstand cyclic loading under the forces and angular displacements seen during gait, ii) has a low enough stiffness to produce negligible stresses at the bone-implant interface, and iii) can be reasonably manufactured. We therefore seek a methodology to optimize for the smallest possible mechanism geometry that hits joint-specific stress and stiffness maximum thresholds during simulated walking gait. Unfortunately, the stress and stiffness behaviors of caged hinge mechanisms are difficult to establish analytically, because of the mechanism’s non-uniform, large deformation; to our knowledge, no analytical model exists for evaluating and designing caged hinges. Finite element analysis (FEA) provides an accurate means of assessing both stress distribution and mechanism stiffness, but the computational overhead makes it difficult to use in an optimization pipeline.

In this manuscript, we present our analysis of the feasibility of compliant intramedullary stems for joint reconstruction. This work falls into the Preclinical Research category of the National Institutes of Health’s Translational Science Spectrum. Specifically, we describe a design process that enables us to generate compliant stem architectures with an expected lifetime of 100 years inside the body. Our approach is built around a neural network that we trained on three-dimensional (3D) FEA data to predict peak stress and mechanism stiffness; this allows us to leverage the accuracy benefits of FEA, without the computational overhead. We used this model to map the relationship between different design parameters and implant performance for three distinct joint applications. This enabled us to generate design spaces for joint-specific stem geometries with a 100-year fatigue life, based on biomechanical and geometric design requirements for both knee and ankle implants. We selected three representative caged hinges from within these design spaces, and additively manufactured a prototype of each; these were then validated experimentally on the benchtop.

## II. Methods and Procedure

### A. Design of Caged Hinge Mechanism

The caged hinge was selected for its ability to support high unidirectional loads while deforming with low stiffness about the loading axis. In designing the gross hinge geometry, our primary goal was to avoid non-fatigue failure modes, including buckling or yielding due to acute overload. To eliminate buckling as a failure mode in the mechanism’s thin blade elements, we incorporated a central post that inverts the caged hinge (Fig. 1) such that it sees a tensile load when the joint is loaded in compression [28]. We also incorporated rigid hard stops that prevent over-rotation of the mechanism, with bounds set at ±12 degrees. This is slightly more than our peak target rotation (Table 1), such that we do not expect the hard stops to engage during most gait activities [29], [30], [31].

**TABLE 1:** Joint-Specific Loading Requirements, Manufacturing Constraints, and Geometric Limits

Anatomic location	Rotational range of motion (deg)	Maximum axial force (N)	Minimum blade thickness (mm)	Intramedullary		Bone-replacing	
				Maximum blade length (mm)	Maximum outer diameter (mm)	Maximum blade length (mm)	Maximum outer diameter (mm)
Distal femur	±10	2415	0.3	85	26.5	63	32.5
Proximal tibia	±10	2415	0.3	72	24	55	30
Distal tibia	±10	4020	0.3	72	22	55	28

We opted to make the caged hinge out of Grade 5 Titanium (Ti64), due primarily to its high yield strength, excellent elastic properties, and biocompatibility [32], [33], [34]. In calculating fatigue life, we assumed 2500 steps per day, or approximately 10^8^ cycles in 100 years. Established stress-number-of-cycles (S-N) curves for Ti64 show an endurance limit (infinite fatigue life) of 410 MPa and a fatigue life of 10^8^ cycles at 483 MPa [35], [36].

The central post that inverts the mechanism (Fig. 1c) is loaded in compression, torsion, and bending due to off-axis forces and moments. We optimized the diameter of this inner post to have a 100-year fatigue life under 6x body weight, with International Organization for Standardization (ISO) standard knee off-axis loads. We then iteratively reduced the diameter of this post until the FEA-predicted stress was below the 100-year fatigue stress of Ti64. This iterative design process resulted in a post with a 12 mm diameter.

### B. FEA Data Collection

We performed an FEA sweep to study the maximum stress and rotational stiffness of different-sized caged hinges under various loads. Five geometric values were selected to parameterize the caged hinge design: blade thickness, blade width, blade length, number of blades, and the radial distance from the center of the caged hinge to the center of the blades (“center distance”). We swept across four of these five parameters, applied linear combinations of tensile load and prescribed angular deformation to each caged hinge geometry (Fig. 1a), and recorded the peak stress within the mechanism and rotational stiffness of the mechanism. To reduce the dimensionality of the FEA sweep, we did not sweep across number of blades because mechanism performance has a predictable relationship with this parameter. The blades act in parallel with each other, such that the number of blades and *tensile* stress are inversely proportional, and the number of blades has no effect on stress from *rotation*. Of note, this assumes that the stems operate only within the linear elastic regime of Ti64, keeping blade stress below the material’s yield strength (950 MPa), and that there are no non-linear stress effects within the blades as a function of load. Because the purpose of this model is to identify mechanisms with a 100-year fatigue life, we are only interested in geometries and loading regimes where the maximum predicted stress is below 483 MPa, which is much below yield and well within the linear elastic regime for Ti64. Mechanism stiffness scales proportionally with the number of blades.

Our objective in performing the sweep was to build a dataset to facilitate the optimization of compliant stems for three distinct applications: the femoral stem of a knee replacement, the tibial stem of a knee replacement, and the tibial stem of an ankle reconstruction. Each intramedullary stem has a unique set of biomechanical design specifications that are fundamental to joint-specific design (Table 1). For both the knee and ankle joints, maximum internal-external angular rotation during walking is based on published biomechanical data [29], [30], [31]. Similarly, published biomechanical data revealed that peak joint reaction forces during walking within the knee and ankle are three-times and five-times body-weight, respectively; we assumed a body weight of 82 kg [15], [37]. To simplify the FEA sweep and complexity of analysis, we chose to focus only on the principle loading axis in this study (uniaxial compression and rotation), and held all other loads at zero.

We considered two clinically relevant stem applications when establishing design requirements (Table 1): true intramedullary stems (inside the bode canal) and bone-replacing stems (such as for megaprostheses). For the intramedullary stems, we set the maximum blade length to 20% of the average length of the bone in which the implant would be housed [38], [39]; we reduced this to 15% for the bone-replacing stems, because added length would require greater resection in those cases [38], [39]. The maximum outer diameter of the bone-replacing femoral stem was set to the mean diameter of the femur at the metaphyseal-diaphyseal transition [40]. The maximum outer diameter of the proximal and distal tibial bone-replacing stems was set to the average diameter of the tibia at 10% of the tibial length from the proximal and distal ends, respectively [41]. The maximum outer diameter of the intramedullary stems was chosen to preserve at least 6 mm of total cortex surrounding the implant [42]. These numbers are not finalized design requirements, as bone size will vary across patients; instead they represent target values when identifying the design space of suitable caged hinge designs.

In addition to biomechanical and anatomical constraints, we established maximum torsional stiffness specifications to ensure that the compliant stem would produce lower shear stress at the bone-implant interface than current stems. Maximum allowable torsional stiffness was calculated for each stem assuming a uniform distribution of stress along the bone-implant interface. Our maximum shear stress threshold was set to 0.1 MPa; this is the *minimum* amount reported for current tibial trays in primary knee replacement [43], [44]. Limitations in both additive and conventional manufacturing required that blade thickness be at least 0.3 mm (Table 1).

The FEA sweep was carried out via an automated pipeline for generating part files, initializing and performing FEA, and extracting peak stress and stiffness results. We first generated part files for 405 caged hinges, with uniform density across the following parameter bounds: length (30-70 mm), width (2-9 mm), thickness (0.3-0.7 mm), and center distance (5-20 mm). Uniform 0.5 mm fillets were applied on the vertices between blades and stages to eliminate stress artifacts and concentrations from our FEA results. FEA was then performed on these part files in ABAQUS (ABAQUS 2019, Dassault Systemes, Velizy-Villacoublay, France). In all FEA, the ground body was rigidly fixed and the stage body was kinematically coupled to a reference point at the center of the free stage along the primary axis of the mechanism. Three iterations of adaptive meshing were then enforced (Fig. 1b), under the maximum applied loads. A concentrated tensile force was applied to the reference point, along the hinge’s primary axis, with a randomly-selected magnitude between 0 and 4020 N (5x body weight). Rotational displacements were then prescribed to the reference point in one degree increments, up to a maximum of ten degrees. Maximum stress and resultant moment at the stage body were extracted from the FEA results. In aggregate, a total of 31,180 stress and stiffness data points were collected from this FEA sweep.

### C. Model Building

On the basis of the FEA results, we explored three models of increasing complexity. Our objectives in this exploration were i) to gain insight into the mechanistic relationships between design parameters and stress within the caged hinge under combined tensile loads and rotational displacement, and ii) to identify a model structure with sufficient accuracy to serve as a framework for future optimization. We present the three models we developed, and provide a characterization of the error landscape associated with each.

#### 1) Analytical Model

We began our modeling efforts by deriving a simple set of analytical equations to calculate peak stress in a caged hinge under combined tension and rotation. For the purposes of this reduced-order model, we assumed that peak stress would occur in the blades, rather than the stage or ground, and could be represented via a Von Mises summation of the stresses from isolated tension under the same load and isolated rotation to the same degree. Note that we did not expect that this simplified model would be sufficiently accurate to support robust peak stress prediction in combined-loading scenarios; however, we hoped that the reduced-order model would prove instructive in understanding how different features of the caged hinge design might affect peak stresses. Derivation of the analytical model is shown in the Appendix.

We used the analytical model to explore the impact of varying each of the primary blade parameters on peak stress within the blades. For each parameter of interest (length, thickness, and width), we generated 10,000 eight-blade mechanism geometries as random combinations of all blade parameters other than the parameter of interest (e.g. when evaluating width, we generated 10,000 combinations of length, thickness, and center distance). For each of these 10,000 geometries, we then swept across the parameter of interest, and calculated peak blade stress under maximum mechanism force (4020 N) and displacement (10 deg). We normalized the stress results from each sweep to the minimum peak-stress value seen for that geometry, and averaged the results together across the 10,000 geometries to produce a generalized relationship between peak stress and each parameter of interest.

**FIGURE 2.**
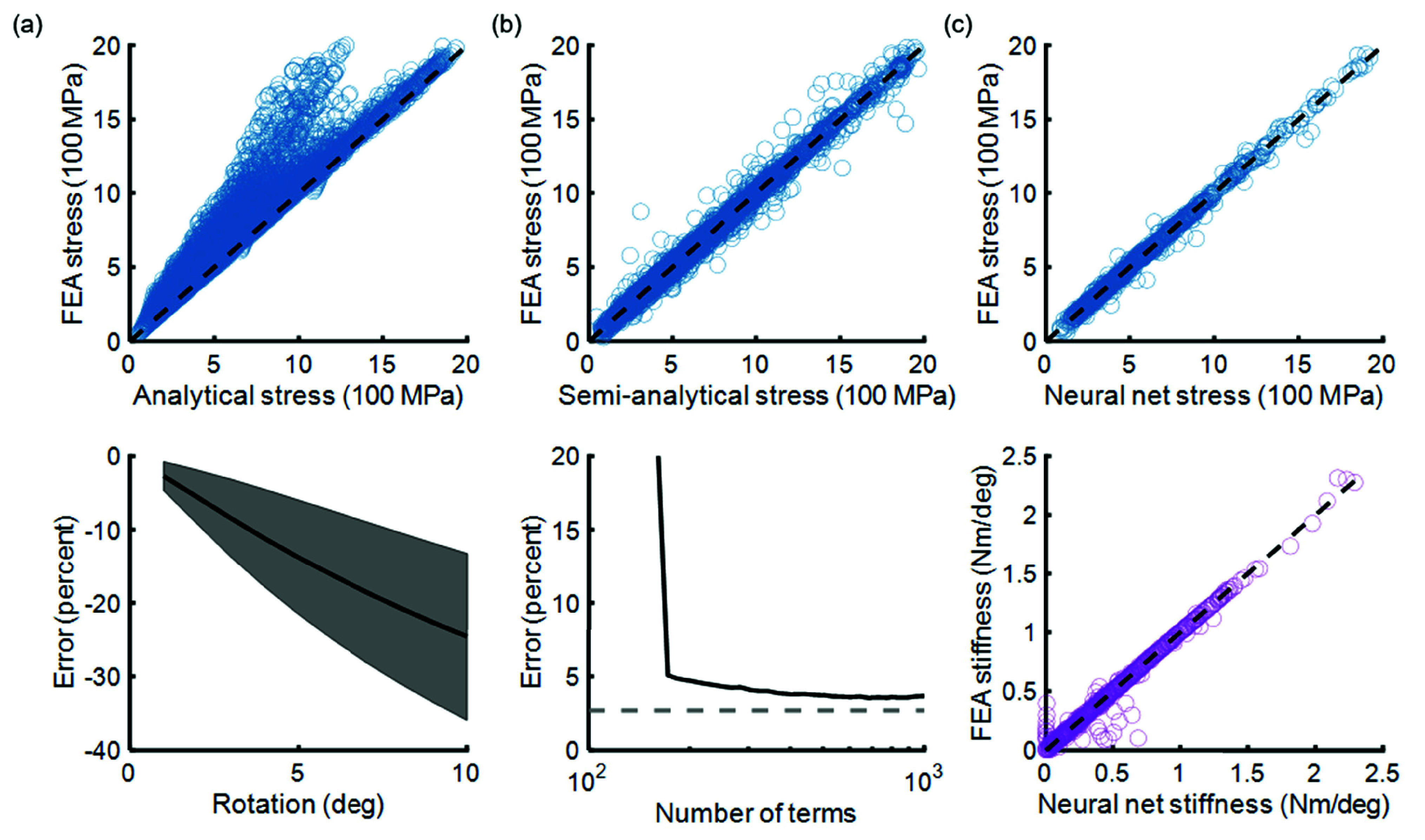
Comparison of models for predicting maximum stress. (a) FEA-simulated stress versus stress predicted by the analytical model. For all model scatter plots, dashed line represents perfect model agreement. Bottom plot shows error between FEA and analytical model as a function of angular displacement (shaded region shows mean ± 1 SD). (b) FEA-simulated stress versus stress predicted by the semi-analytical model (top). Error of the semi-analytical model versus number of terms in the model (bottom). (c) FEA-simulated versus neural-network-predicted stress (top) and stiffness (bottom).

To confirm our suspicions that the superposition assumption inherent to this analytical model would break down under large displacements, we cross-checked peak stress predictions from this model against the results of the FEA sweep. Unsurprisingly, we found that the analytical model becomes less accurate as deformation increases, when compared to 3D FEA (Fig. 2a). The average absolute error across the full range of simulated geometries, loads, and displacements was 8.8%. However, the analytical model systematically under-predicts peak stress, by as much as 15-20% at full angular displacement. This systematic and highly variable inaccuracy would make the model difficult to use in optimizing caged hinge geometry for a given loading environment.

#### 2) Semi-Analytical Model

The goal of our semi-analytical approach was to see whether a simple algebraic expression for the data could be inferred by iteratively regressing the data with a large set of possible explanatory terms, and eliminating less-significant terms in each iteration. Details for creation and evaluation of the semi-analytical model are included in the Appendix.

We estimated error as a function of number of terms by cross-validation (70% to training, 30% to testing) from the complete FEA dataset (31,180 points). Mean absolute percent error for the model saturated at 3.7% around 500 terms (Fig. 2b). As terms are subtracted, error increases, with a sharp jump below about 168 terms, suggesting that a large number of terms is needed to capture the underlying physical phenomena. No overfitting was observed in the cross validation. These simulations suggest that there is likely not a simple algebraic expression that captures the maximum stress as a function of our baseline explanatory variables (geometric parameters, load, and angular displacement), to useful accuracy for engineering purposes.

#### 3) Neural Network

We trained neural networks to predict maximum stress and torsional stiffness from i) caged hinge geometry, ii) tensile force magnitude, and iii) angular displacement. The neural networks were constructed with MATLAB’s Deep Learning Toolbox (MATLAB 2021, MathWorks, Natick, MA, USA). During model tuning, the complete dataset (31,180 points) was split randomly into three subsets: 70% went to training, 15% to validation, and 15% to testing. Absolute error was computed with respect to FEA data and averaged over all points in the test set. Each network architecture was tuned iteratively, adding additional layers or nodes to increase accuracy while avoiding overfit by comparing the average error of the training set with the average test set error. The final architecture of the stress neural network was one layer with 180 nodes, and the architecture of the stiffness network was one layer with 100 nodes. Cross-validation was performed over ten trials to ensure consistency in error findings; the average relative errors across all ten cross-validation trials were 2.69% and 4.09% for stress and stiffness respectively. Once the neural network parameters were tuned, final models for stress and stiffness were trained with all available data (Fig. 2c).

### D. Exploratory Simulation

We used our models to investigate the relationships between key geometric parameters of the caged hinge and both maximum stress and torsional stiffness. We were most interested in the effects of blade length, thickness, and width because these are the three parameters that can most easily be manipulated in designing a joint-specific intramedullary stem. We explored how peak stress and stiffness were affected by each of these key parameters in isolation, as well as how peak stress and stiffness varied across two-dimensional landscapes of these parameters. For each analysis, all parameters except the parameter(s) of interest were held fixed at nominal values, and stress/stiffness were assessed under the peak compressive load and deformation from the proximal tibia. The number of blades was held constant at 12. Center distance was set for each geometry based on blade width, such that the distance from the inner edge of the blades to the center of the mechanism was fixed at 7 mm; this ensured that all geometries we evaluated would be able to accommodate a post of up to 14 mm in diameter.

We also used the neural network to generate a “design space” for each application, constituting a boundary—within a two-dimensional co-varying parameter space—of stem geometries with a 100-year lifetime. To convert stress to lifetime, we assumed the patient would take an average of 2500 steps per day and applied the worst-case loading scenario for each cycle: maximum ISO tensile force at full 10 deg rotation. In making the design space for each anatomic location, the x- and y-axis variables were swept while all other geometric parameters were held constant at the values chosen for manufacturing. From these design spaces, we selected three mechanism designs (one for each anatomic location) to manufacture and validate on the benchtop.

### E. Benchtop Validation

Three caged hinge prototypes were 3D printed in Ti64 for experimental validation (Protolabs, Inc., Maple Plain, MN, USA). We also manufactured a single case that was compatible with all three hinges, to demonstrate viability of the complete stem system (PartsBadger, LLC, Cedarburg, WI, USA). Unfortunately, imperfections in manufacturing caused the hinges to rub on the walls of the case when loaded (SM Movie 1). To resolve this, we opted to evaluate each hinge mechanism in isolation, without the case. Benchtop evaluation of the caged hinges was conducted on a six degree-of-freedom serial manipulator (KUKA, Augsburg, Germany). Each hinge was attached on one end to the KUKA’s flange, and on the other end to a grounded base. Loading was carried out in two steps: a tensile load was first applied, and then the flange side of the mechanism was rotated by ten degrees with respect to the base, and then rotated back to the initial position (SM Movie 2). This was repeated ten times. The mechanism was then unloaded, and the process repeated for a different tensile load. Tensile loads were systematically increased from 0 to 2000 N, in 500 N increments. The maximum tensile force was limited by the measurement range of the KUKA’s load cell. Point kinematics and six-axis forces and moments were recorded and used to calculate rotational stiffness of the mechanism under each load.

Once each mechanism had been characterized, we pushed the distal femoral hinge to failure. To do this, we loaded to 2000 N in tension, and then rotated the flange by 150 degrees relative to the base, while recording displacement and six-axis forces and torques. We then removed the tensile load from the mechanism and rotated back to the initial position. As a last step, we reapplied the 2000 N of tension to the deformed device (SM Movie 3).

## III. Results

### A. Trends in Stress and Stiffness

**FIGURE 3.**
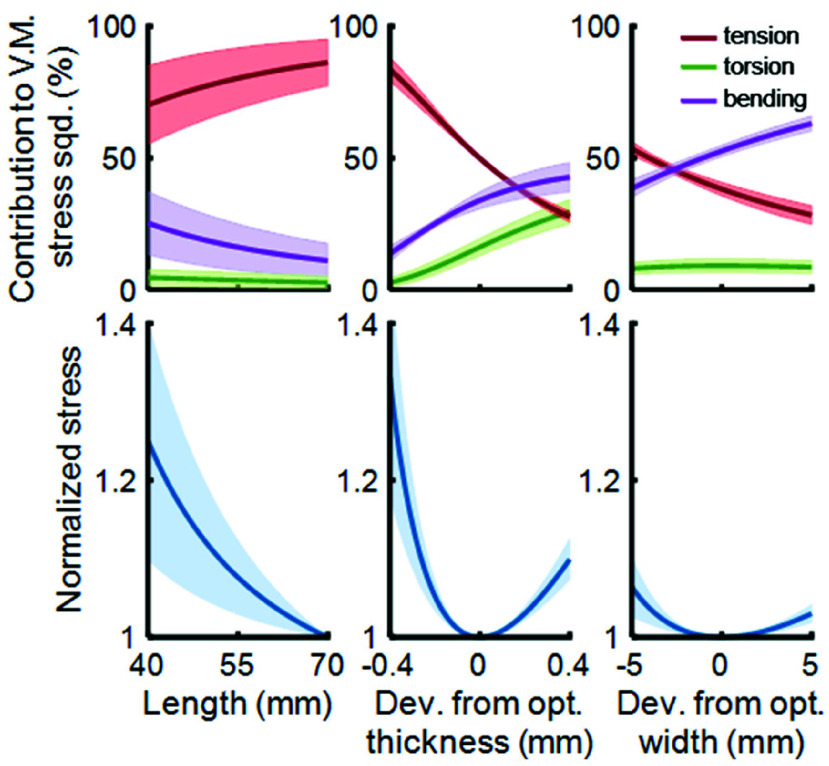
Stress trends in the analytical model. Each geometric parameter was swept independently over 10,000 random geometries. Top row plots the contributions of tensile, bending, and torsional stress to the Von Mises stress (shaded region is mean ± 1 SD). Bottom row plots normalized stress as a function of geometric parameters. For each of the parameters of interest (x-axis values, e.g. length), all other parameters were held fixed at randomly generated “base geometry” while the parameter of interest was swept; stresses from each such sweep were normalized to the minimum stress for that base geometry. This was then repeated across 10,000 base geometries, and the average normalized stress was plotted as a function of the parameter of interest (shaded region is mean ± 1 SD).

We developed the analytical model with the intent of deconstructing key trends in our peak stress predictions and explaining those trends as a function of the underlying mechanics. Toward that end, the analytical model predicts a monotonic decrease in peak stress as blade length increases, driven primarily by reductions in the torsional and rotational components of total stress (Fig. 3). This phenomenon is consistent across blade geometries, although there is substantial variance in the slope of this relationship. The relationship between peak stress and blade thickness is consistently parabolic, with large increases in peak stress as the blade becomes either too thin to handle the tensile loads, or too thick to support the bending and torsional elements of mechanism rotation. A similar relationship exists between peak stress and blade width, albeit much less dramatic. This would refute the idea that a larger mechanism is always better; in fact, as the blades get wider with a fixed center distance, the distance traveled by the outer edge of the blades increases proportionally, driving up the bending stress. As the blade widens, this increase in bending stress eventually overtakes any reduction in stress from axial tension that would come from a larger blade cross section, such that total peak stress increases.

**FIGURE 4.**
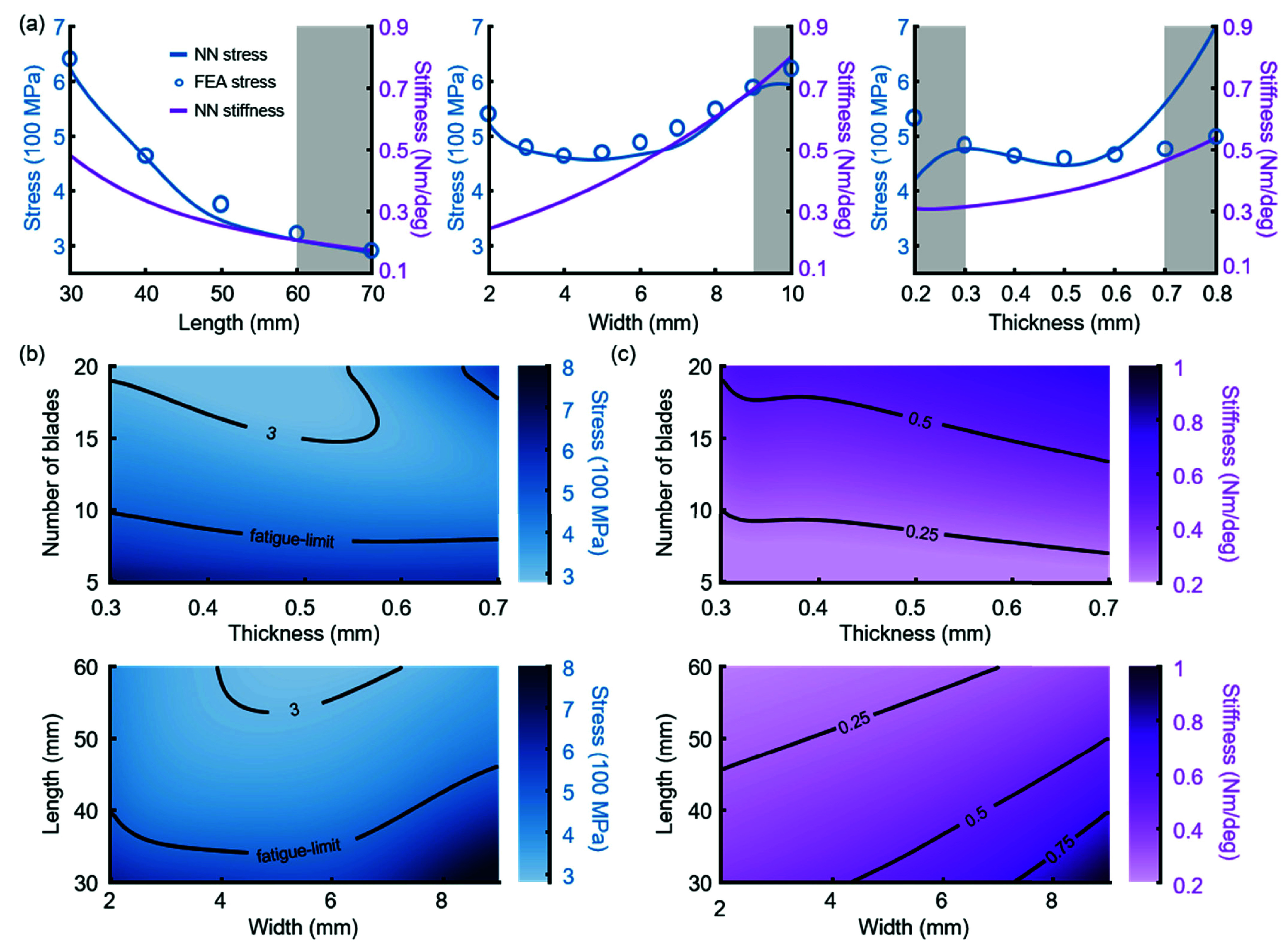
Stress and stiffness trends in the neural network. (a) Maximum stress and torsional stiffness predicted by the neural net (NN) versus blade length, width, and thickness. Gray backgrounds represent areas of model extrapolation. Note that the lines on these plots are cross-sectional cuts from the heatmaps in (b) and (c). FEA-simulated stress values points are overlayed for comparison. (b) Heatmap of maximum stress as a function of geometric parameters. All geometries within the fatigue limit (483 MPa) contour are expected to have at least a 100-year fatigue life in the body. (c) Heatmap of stiffness as a function of geometric parameters. For (a), (b), and (c) each variable of interest was independently swept while all other parameters were held constant at length (40 mm), width (4 mm), thickness (0.4 mm), inner diameter (14 mm), number of blades (12), axial force (2415 N), and angular displacement (10 deg).

These stress trends were reflected in the neural network’s predictions and were evident in the source FEA data. (Fig. 4). The stiffness neural network predicts that torsional stiffness decreases with increasing blade length, is proportional to blade width, and increases with increasing blade thickness (Fig. 4a). We also found that performance of the neural networks drops off dramatically when the network is used for extrapolation beyond the bounds of the training set (Fig. 4a, gray region). In two dimensions, we observed that the convexity in stress as a function of thickness is preserved across different blade counts, but that the location of the minimum-stress thickness varies with blade count (Fig. 4b). Additionally, adding more blades always produces lower stresses, but increases mechanism stiffness. Similarly, the minimum-stress thickness varied as a function of blade length. Increasing blade length—and thus mechanism envelope—always increases performance (lower stress, lower stiffness) at the minimum-stress width (Fig. 4b,c).

### B. Design Spaces

**FIGURE 5.**
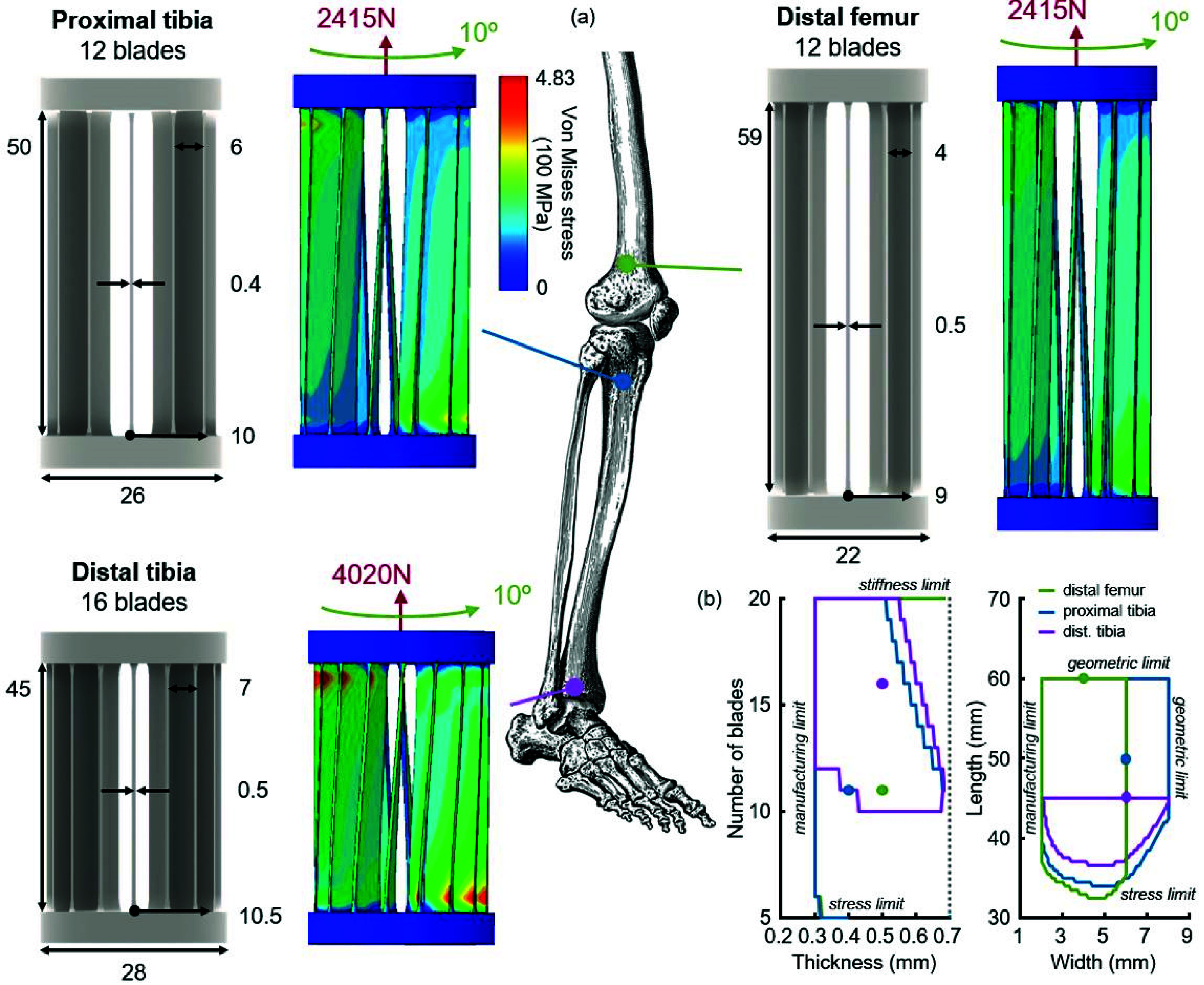
FEA validation of the prototype stems as manufactured. (a) Geometric parameters selected for the three manufactured caged hinges (all values in mm). FEA results of as manufactured hinges under joint-specific combined loading. (b) Design spaces for all three anatomic locations. Contours encompass all caged hinge geometries with a 100-year fatigue life under their joints loading profile. Dots show the parameters that we selected for manufacturing. For each anatomic location, the variables of interest were swept while all other geometric parameters were held constant at the values chosen for manufacturing.

We were able to identify sizeable design spaces of stem geometries with a predicted 100-year fatigue life for each of the three anatomic locations (Fig. 5). The boundaries of these design spaces were derived from stiffness limits, geometric envelopes, manufacturing constraints, and fatigue stress. Specifically, the number of blades was limited for all stems on the high end by stiffness limits and on the low end by fatigue stress. Blade thickness was limited on the low end by either a manufacturing constraint or, for lower blade counts, fatigue stress. For the distal femoral stem, we did not reach an upper bound on thickness without extrapolating beyond the bounds of the neural net; however, our heatmaps did show that the minimum-stress thickness lies within the model bounds. For the other two anatomic locations, at high blade counts blade thickness was limited by our stiffness limits. Length and width for all three mechanisms were limited on the high end by the maximum geometric envelope. The minimum width was set as a manufacturing constraint, and minimum length was limited by fatigue stress. The distal tibial mechanism had the smallest overall design space, limited primarily by tighter length restrictions and higher axial loads relative to the other two anatomic locations.

From each of the three design spaces, we selected one mechanism geometry to manufacture for experimental validation (Fig 5b, dots). Note that the geometries we selected were not necessarily “optimal”, as identifying true optimality would require a cost function that balances both geometry and performance (e.g. a smaller mechanism might have higher-but-still-feasible stresses than a larger mechanism, but be more “optimal” because it is smaller). Instead, the selected mechanism geometries represent arbitrary feasible designs from within each design space. Noting significant imperfections in our prototypes, we measured each blade thickness of the *as manufactured* prototypes, and updated our digital models accordingly. FEA-predicted peak stresses for these three caged hinges *as manufactured* were below the fatigue limit under application-specific loading and deformation (Fig. 5a). The modeled stresses the caged hinges would experience under peak axial loads at 12 degrees of rotation were below Ti64’s yield stress [34].

### C. Benchtop Validation

**FIGURE 6.**
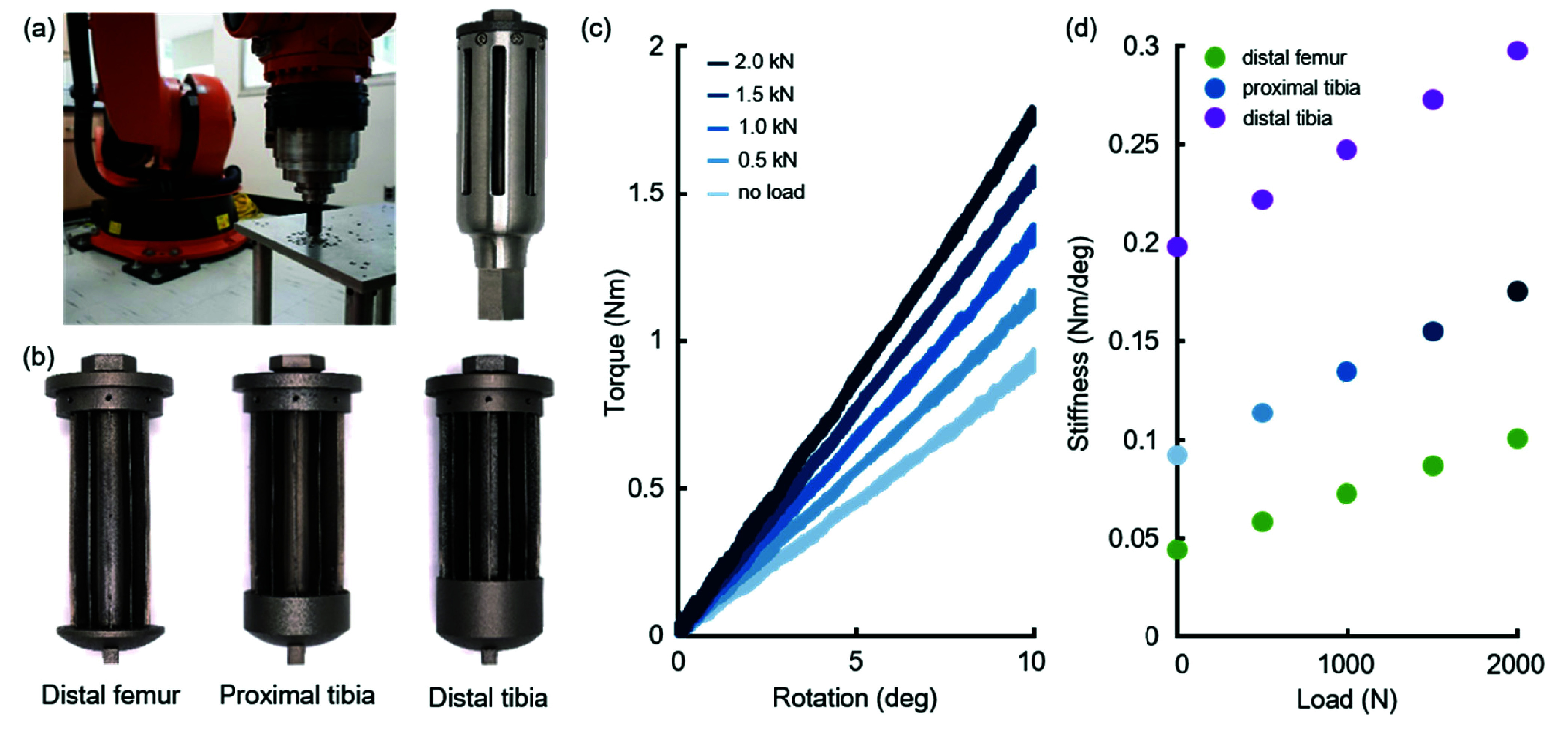
Manufactured caged hinges and experimental results. (a) Experimental setup with the KUKA robot and caged hinge, and a caged hinge assembled with the case. Torque and angle were measured on a load cell mounted into the KUKA arm. (b) Three additively manufactured caged hinges. The blades of the distal femoral and proximal tibial caged hinges experienced noticeable bowing (blades are thinner at the ends and wider in the middle). (c) Torque versus angle for the proximal tibial caged hinge at different loads; raw data from all ten trials for each load case shown on plot. (d) Stiffness at different loads for all three caged hinges. Stiffness values for each case represent the slope of the best-fit line to all torque-angle data for that hinge and load. Blue dots shaded to match line corresponding lines in (c).

All three prototypes were validated on the KUKA robot (Fig. 6a). The 3D printed prototypes *as manufactured* had notable imperfections, including warping, blade defects, and variations in blade thickness (Fig. 6b). However, the gross mechanism geometry was deemed sufficiently similar to the target design to capture trends in mechanism behavior. Our benchtop results showed a linear relationship between deformation angle and rotational torque, with no notable hysteresis (Fig. 6c). Rotational stiffness increased with tensile load in all three prototypes (Fig. 6d). The peak measured stiffnesses of all three mechanisms were below the maximum stiffness limits we established for each anatomic location.

**FIGURE 7.**
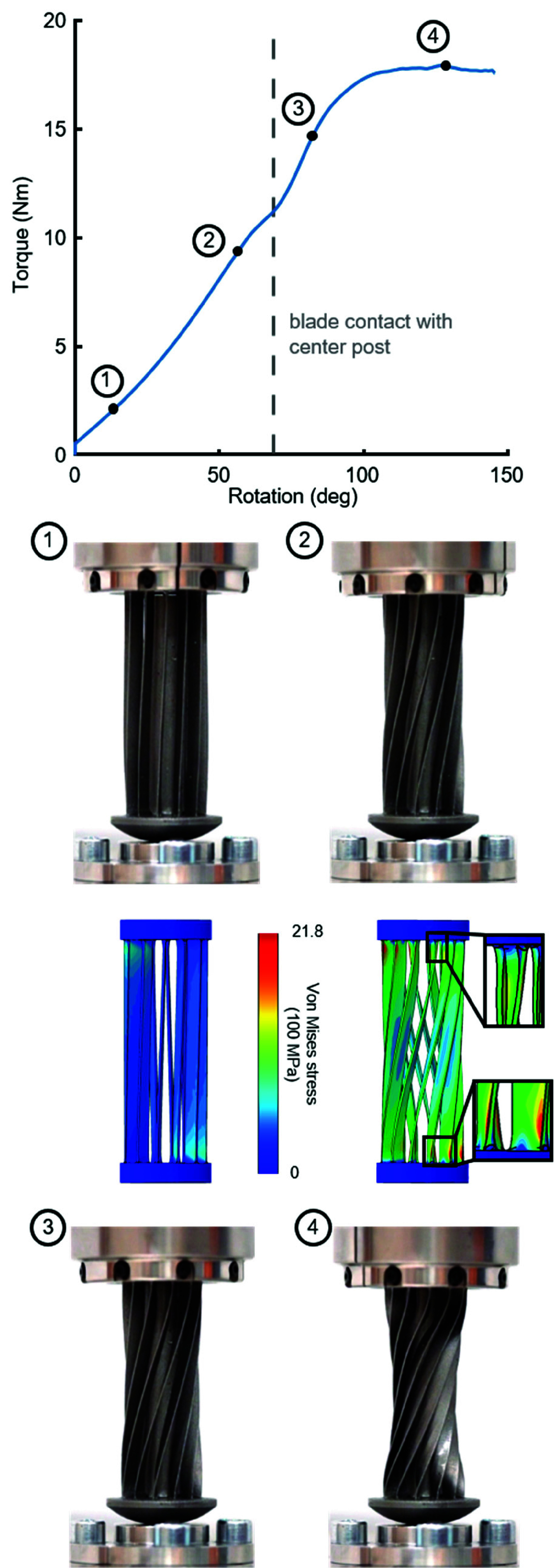
Failure evaluation of the distal femoral caged hinge, under a combined loading of 2000 N and 150 degrees of rotation. Vertical dashed line denotes where the blades first contact the post. Images of the caged hinge at various points along the torque-angle curve. For comparison, FEA shown for the loading conditions at points 1 and 2.

For the mechanism pushed to failure, the torque-angle relationship remained linear until yield, which occurred around 60 degrees (Fig. 7, point 2). After yield, we observed a decreased stiffness consistent with plastic deformation, until around 68 degrees of rotation. At this point, the blades contacted the post (Fig. 7, point 3), causing the mechanism to stiffen again until failure, which occurred around 100 degrees (Fig. 7, point 4). FEA-predicted stresses for the *as manufactured* hinge under the loads at which we observed yield were well above the material’s yield stress. Geometric predictions of blade deformation were remarkably consistent with the observed behaviors, including small regions near the blade ends with local buckling (Fig. 7, FEA callouts).

## IV. Discussion

In this manuscript, we presented and validated a novel compliant intramedullary stem architecture with the potential to simultaneously address both aseptic loosening and over-constraint. We implemented an analytical model and neural networks to study how the mechanism’s geometry affects its performance. The neural networks informed our design of viable compliant stems for three different anatomical locations. We created prototypes of these three stems and established on the benchtop that they function as designed.

Our modeling results indicate that there are complex non-linear relationships between mechanism geometry and peak stress under combined loading and angular displacement. The analytical model rapidly became unreliable as displacement increased; although the model was helpful in explaining the observed trends, the error was too large for the model to be useful in design. Accuracy of the semi-analytical model increased sharply once the model reached around 168 terms, and then slowly converged to an average error around that of the neural network as more terms were added, suggesting that there is not a simple algebraic expression that captures the maximum stress as a function of our input variables. The neural network was quite accurate within the parameter bounds of the training set; however, accuracy quickly fell off for geometries that extrapolated beyond these bounds (Fig. 4a). Training the neural network required a large data set, generated over weeks of simulation; this computational overhead will only be compounded as we seek to increase parameter ranges or add additional features to the model.

Stress always decreases with increased blade length and number of blades. For a fixed length, number of blades, and inner diameter, there exists both a minimum-stress thickness and width. If the design goal is strictly to minimize stress, the post diameter should be minimized as much as possible while still supporting the required loads, and both length and number of blades should be maximized within geometric limits and manufacturing constraints. From there, the thickness-width space should be swept for an optimal minimum-stress point. Minimum-stiffness optimization will always prioritize increased length, and decreased width, thickness, and number of blades.

Our analysis revealed a large parameter space of feasible caged hinge geometries, with peak stresses below Ti64’s fatigue limit and stiffnesses below our maximum allowable stiffness. This indicates that there is room for the stem mechanisms to be designed with a safety margin to accommodate off-axis moments or acute overload. Once an appropriate safety margin has been established, stem volume can also be reduced. When optimizing volume, inner diameter should be minimized and the number of blades maximized, and from there an optimal point should be found from within the length-width-thickness space. It is clear from our length-versus-width design space that there is a direct tradeoff in performance between these two parameters (which govern overall volume). This tradeoff presents an opportunity to design patient-tailored stems; overall length and outer diameter can be exchanged to find an optimum based on the patient’s anatomy (e.g. short, squat or long, thin bones).

Manufacturing of the prototype stems for our experimental validation posed significant challenges. The 3D printed caged hinges had noticeable manufacturing defects, which were likely significant enough to affect their mechanical performance. In the future, these stems will be cut on a rotary wire EDM, which will provide much more precision than 3D printing, albeit with an increased cost and time delay. The gross behaviors of the caged hinges *as manufactured* were consistent with our simulations: there was a linear torque-angle relationship, and stiffness increased with increasing load. This latter behavior is a function of a parasitic screw motion of the stage relative to the grounded body during large rotations: with increased axial loads, there is more resistance to this parasitic translation of the stage along the hinge’s long axis. This means that the bone-implant interface would experience more shear stress under higher joint loads; stem design should therefore be optimized in consideration of stiffness under the *highest* expected repetitive joint loads.

Despite manufacturing defects in our prototypes, yielding occurred at much higher deformation than our FEA predicted. One possible explanation is that the 3D printed titanium had a higher-than-reported yield strength; reported properties of 3D printed metals are often imprecise, and depend on specifics of the build parameters. Another possibility is that the device actually did yield as predicted, but this yielding happened very locally in isolated pockets (Fig. 5), and did not cause grossly observable changes in the torque-angle relationship. In the failure experiment, the stem was able to rotate 150 degrees under a 2000 N load without failing catastrophically. This behavior appears to have been aided by the post, which served as a hardstop of sorts to prevent buckling of the blades. A lack of catastrophic post-yielding behavior is an important feature for an implant because catastrophic failure within a joint could be dangerous. In practice, the device would never see this much deformation; hardstops in the case would prevent this degree of over-rotation.

To reign in the simulation requirements for this manuscript, we opted not to include off-axis loading of the stem in the models. Moments about axes other than the stem’s long axis and forces orthogonal to the stem’s long axis would increase stresses in the blades and could even induce buckling behaviors. However, because axial forces represent the dominant load experienced by intramedullary stems, we do not expect that the addition of relatively small off-axis loads will significantly alter the predicted stresses at maximum loading. We were also conservative in our *uniaxial* loading estimates, using the worst-possible loading conditions (maximum compressive loading and maximum angular deformation) for our analysis; it is likely that this substantially overestimates the stress that the blades will see during cyclic gait, where these two conditions do not typically coincide. Our final implant design will be optimized in the context of six degree-of-freedom loads. The device will also include polyethylene hardstops to offload the central caged hinge in overload scenarios; although these hardstops introduce the potential for wear, they will be designed to engage only when necessary to protect the central mechanism, with a fraction of the loading frequency of conventional rotating platforms.

The purpose of this work was to establish a framework for the design of compliant stems. This represents an important translational step toward intramedullary and bone-replacing stems that accommodate natural joint motion. Future work will involve using the tools presented here to produce optimal stem designs for specific clinical applications. We will also perform long-term fatigue testing to validate the lifetime of the optimized stems, assess the biocompatibility of the mechanism, and experimentally characterize interfacial stresses and micromotion at the bone-implant interface in cadaver limbs. Prior to moving into clinical trials, we will also formalize a sustainable manufacturing pipeline for these compliant stems, and obtain all necessary regulatory approvals. Although we only considered stem designs for knee and ankle joint reconstruction in this proof-of-concept study, our predictive models could also be applied directly to the design of intramedullary stems for all joint arthroplasty, such as for the hip, elbow, and wrist. Because these stems are modular, we expect that they could be seamlessly introduced into current and future joint replacements.

## V. Conclusion

Herein we present the design, modeling, and validation of compliant intramedullary stems that address the primary non-infectious causes of joint replacement revisions worldwide [8], [9], [10], [11], [12]. Our compliant stems accommodate more rotation than is seen in either the biological knee or ankle during gait without introducing articulating loaded surfaces. The compliant mechanism we designed is unique in its ability to guide rotational stem motion within the body without creating friction, which has the potential to reduce aseptic loosening and prolong implant longevity across many different joints and pathologies.

## Supplementary Materials

Supplementary materials

## Appendix

### A. Analytical Model Formulation

Normal stress from tension was modeled as a uniaxial point force acting uniformly the blade’s cross section, along the blade’s primary axis:

\begin{equation*} \sigma _{Tens}=\frac {F_{T}}{wt} \tag{1}\end{equation*}

where $F_{T}$ is the force applied per blade (mechanism tensile force divided by number of blades), $w$ is blade width, and $t$ is blade thickness.

Rotation of stage relative to the base creates both a torsion about the blade’s long central axis and a bending as the blade accommodates the stage’s arcuate displacement. In estimating peak stress, we considered each of these effects separately, but assumed that the peak stresses from both effects were co-located on the blade. Peak shear stress from torsion for a fixed angular displacement should occur at the corners of the blade’s rectangular cross section. Peak normal stress from bending for a fixed mechanism angular displacement should occur along one of the blade’s flat faces, in the portion of the blade that deflects the most: in this case, the outer blade edge, which has to accommodate a larger arc length than the rest of the beam for a fixed angular displacement of the mechanism. These two areas of peak stress intersect along the outside corner of the blade, which is where we would expect to find the maximum overall stress in the beam (Fig 5a).

In torsion, the blade was considered to be a beam of rectangular cross section, twisted to a fixed angular displacement equal to the rotational displacement of the full mechanism. Established equations for torsion of a rectangular beam tell us that:

\begin{align*} \tau _{Tor\mathrm {, }max}&=\frac {T}{\alpha wt^{2}} \tag{2}\\ \theta _{rot}&=\frac {TL}{\beta wt^{3}G} \tag{3}\end{align*}

where $\theta _{rot}$ is the angular displacement, $T$ is the torque acting to twist the beam, $L$ is the length of the beam, $G$ is the material’s shear modulus, and $\alpha $ and $\beta $ are geometric parameters related to the beam’s cross section (specifically, the ratio of blade width to blade thickness).

We can then solve (2) for $T$ and substitute into (3), which provides us an expression for maximum torsional stress in the beam as a function of angular displacement:

\begin{equation*} \tau _{Tor\mathrm {, }max}=\frac {\beta }{\alpha }\frac {Gt}{L}\theta _{rot} \tag{4}\end{equation*}

In bending, the blade was modeled as a fixed-guided beam, displaced at one end by the arc length traversed by the blade end as the mechanism rotates. Because the maximum stresses occur in the part of the blade that experiences the largest displacement, the arc length was calculated based on the distance from the center of the mechanism to the *outer edge* of the blade:

\begin{equation*} \delta =\left ({R_{center}+\frac {w}{2} }\right)\theta _{rot} \tag{5}\end{equation*}

where $R_{center}$ is the distance from the center of the mechanism to the center of the blade (“center distance”).

Established equations for bending of a fixed-guided beam tell us that:

\begin{align*} \sigma _{Bend\mathrm {, }max}&=\frac {FL\frac {t}{2}}{2I} \tag{6}\\ \delta &=\frac {FL^{3}}{12EI} \tag{7}\end{align*}

where $\delta $ is the linear displacement, $F$ is the force at the end of the beam perpendicular to the beam’s long axis, $E$ is the material’s elastic modulus, and $I$ is the second moment of area.

Solving (6) for $F$ and substituting into (7) provides an explicit expression for maximum bending stress as a function of linear displacement:

\begin{equation*} \sigma _{Bend\mathrm {, }max}=\frac {3Et}{L^{2}}\delta \tag{8}\end{equation*}

Substituting $\delta $ for our arc-length calculation from (7):

\begin{equation*} \sigma _{Bend\mathrm {, }max}=\frac {3Et}{L^{2}}\left ({R_{center}+\frac {w}{2} }\right)\theta _{rot} \tag{9}\end{equation*}

Superposing these component stresses, we can generate an expression for maximum Von Mises stress in each blade:

\begin{align*} &\hspace {-.1pc}\sigma _{VM,max} \\ &=\sqrt {\left ({\sigma _{Tens}+\sigma _{Bend, max} }\right)^{2}+3\tau _{Tor, max}^{2}} \tag{10}\\ &\hspace {-.1pc}\sigma _{VM,max} \\ &=\sqrt {\left ({\frac {F_{T}}{wt}+\frac {3Et}{L^{2}}\left ({R_{center}+\frac {w}{2} }\right)\theta _{rot} }\right)^{2}+3\left ({\frac {\beta }{\alpha }\frac {Gt}{L}\theta _{rot} }\right)^{2}} \tag{11}\end{align*}

### B. Semi-Analytical Model Formulation

To build the set of candidate explanatory terms for the semi-analytical model, we started with seven baseline explanatory variables ( $L$, $t$, $w$, $R_{center}$, $n_{blades}, F_{T}$, and $\theta _{rot}$) and augmented them by adding four terms from the analytical model: $t\frac {\theta _{rot}}{L}$, $\frac {F}{wt}$, $\frac {tR_{center}\theta _{rot}}{L^{2}}$, and $t\left ({\frac {w}{2} }\right)\frac {\theta _{rot}}{L^{2}}$.

We then took this augmented set of eleven explanatory terms and further augmented them with the inverses of nine of those eleven (excluding $F$ and $\frac {F}{wt}$, which both included zero entries). To these we added positive powers of $F$ up to $F^{6}$. We then expanded the resulting augmented set of 25 explanatory terms by computing all of their multivariable monomial products up to cubic powers. This resulted in 8,436 candidate explanatory terms, including a few duplicates resulting from the inverses and powers added prior to polynomial expansion. These candidate explanatory terms were arranged into an n x 8,436 matrix, where n was the number of datapoints, with low-order terms in the leading columns.

The subtractive model selection process proceeded cyclically as follows. We calculated the QR decomposition of the data matrix to orthonormalize the data; one important property of the QR decomposition is that the i’th columns of the Q matrix is a linear combination of the leading i columns of the data matrix, and can be thought of as the increment of new information added by the i’th column relative to the leading i-1 columns. We then calculated the linear regression coefficients of the maximum stress as a function of Q. Because Q is orthonormal, we could directly compare regression coefficients between variables in the regression; so for each iteration we dropped some fraction of the columns of the data matrix that corresponded to the terms with the lowest absolute regression coefficients. This process repeated until only a single term remained in the model.
